# Basophils contribute to pristane-induced Lupus-like nephritis model

**DOI:** 10.1038/s41598-017-08516-7

**Published:** 2017-08-11

**Authors:** Barbara Dema, Yasmine Lamri, Christophe Pellefigues, Emeline Pacreau, Fanny Saidoune, Caroline Bidault, Hajime Karasuyama, Karim Sacré, Eric Daugas, Nicolas Charles

**Affiliations:** 1 Centre de Recherche sur l’Inflammation, INSERM UMR1149, CNRS ERL8252, Université Paris Diderot, Sorbonne Paris Cité, Faculté de Médecine site Bichat, Laboratoire d’Excellence Inflamex, DHU FIRE, Paris, France; 2 Department of Immune Regulation, Graduate School of Medical and Dental Sciences, Tokyo Medical and Dental University (TMDU), Tokyo, 113-8510 Japan; 3 Department of Internal Medicine, Faculté de Médecine site Bichat, DHU FIRE, Paris, France; 4 Department of Nephrology, Hôpital Bichat, Assistance Publique-Hôpitaux de Paris, Université Paris Diderot, Faculté de Médecine site Bichat, DHU FIRE, Paris, France

## Abstract

Lupus nephritis (LN), one of the most severe outcomes of systemic lupus erythematosus (SLE), is initiated by glomerular deposition of immune-complexes leading to an inflammatory response and kidney failure. Autoantibodies to nuclear antigens and autoreactive B and T cells are central in SLE pathogenesis. Immune mechanisms amplifying this autoantibody production drive flares of the disease. We previously showed that basophils were contributing to LN development in a spontaneous lupus-like mouse model (constitutive *Lyn* ^−/−^ mice) and in SLE subjects through their activation and migration to secondary lymphoid organs (SLOs) where they amplify autoantibody production. In order to study the basophil-specific mechanisms by which these cells contribute to LN development, we needed to validate their involvement in a genetically independent SLE-like mouse model. Pristane, when injected to non-lupus-prone mouse strains, induces a LN-like disease. In this inducible model, basophils were activated and accumulated in SLOs to promote autoantibody production. Basophil depletion by two distinct approaches dampened LN-like disease, demonstrating their contribution to the pristane-induced LN model. These results enable further studies to decipher molecular mechanisms by which basophils contribute to lupus progression.

## Introduction

Systemic Lupus Erythematosus (SLE) is a complex and heterogeneous autoimmune disease characterized by the production of antibodies against self-antigens mostly of nuclear origin such as double-stranded DNA and ribonucleoproteins (RNP). These autoantibodies form pathogenic immune-complexes (ICs) once aggregated to their autoantigens and complement factors^1^. Defects in B/T cell tolerance promote the uncontrolled accumulation of such ICs that interact with cells bearing Fc receptors leading to chronic inflammation and organ damage^2, 3^. When glomerular deposition of ICs and complement activation occur, a consequent inflammatory response is initiated leading to kidney damage, known as Lupus Nephritis (LN). LN is considered a chronic kidney disease and is one of the most severe outcomes in SLE affecting 30 to 60% of patients^2^. The study of different SLE-like mouse models to decipher the underlying immunological processes that lead to this kidney failure has helped to progress in the development of new therapeutic strategies^3^.

Basophils, known to be the rarest blood-circulating granulocyte, are well described to play important roles in allergic inflammation and protective immunity against parasitic infections, through their expression of high affinity IgE receptor (FcεRI)^4^. But the extensive research in basophil biology has broadened our understanding of new and more crucial regulatory competences in different immunological settings^5^. Indeed, basophils have been characterized to interact with other cell types^5^, being part of the complex network of some inflammatory responses. In normal conditions and in a lupus-like environment, basophils cooperate with T and B cells to enhance proliferation, expansion and differentiation of antibody producing cells^6–9^, through their ability to express surface markers (BAFF, MHC-II) and to secrete cytokines (IL-6, IL-4)^6–10^. Previously, we demonstrated that basophils were activated and accumulated in SLOs in an IgE and IL-4 dependent manner during lupus-like disease development where they were supporting CD19^+^CD138^+^ short lived plasma cells to amplify autoantibody production^6, 11^. However, the genetic deficiencies in the spontaneous lupus-like mouse models used (*Lyn* ^−/−^, *FcγRIIB* ^−/−^, *FcγRIIB* ^−/−^ *Yaa*)^6, 11^ may have contributed to this phenomenon. Indeed, basophil hyper-activation is at least partially due to intrinsic defects (Lyn, a Src family kinase known to regulate several immunoreceptor signaling; or FcγRIIB (CD32B), an inhibitory receptor with low affinity for Fc portion of IgG) which are difficult to discriminate from other cell types having as well aberrant physiologies^12–14^. In order to be able to study mechanisms by which basophils contribute to LN pathogenesis independently of genetic deficiencies, we aimed to determine their contribution to an inducible non-genetic lupus-like mouse model.

Pristane (TMPD; 2, 6, 10, 14- tetramethylpentadecane) is an isopranoid alkane, a component of plants and mineral oils. When pristane is injected in the peritoneum of a non-prone autoimmune mouse strain, a chronic inflammation develops, generating lipogranulomas and ectopic lymphoid tissue in the peritoneal cavity. The adjuvant properties of pristane induce an increased availability of nuclear antigens leading to an autoantibody production^15^. Pristane then generates the pro-inflammatory milieu to have a lupus-like disease development, resulting in an IC mediated glomerulonephritis^16, 17^. This inducible model of lupus-like disease consists of a single intraperitoneal (ip) injection of pristane in 8 weeks-old female mice. Lupus-like parameters have been described as being measurable 24 weeks (*ie* 6 months) after pristane injection in both Balb/c and C57BL/6 mice^16, 18^. This pristane-induced lupus–like disease is cytokine driven, with a strong type I IFN signature and a TLR7-mediated autoantibody production^19–21^. These features make this lupus-like disease one of the lupus-models which fulfills the more criteria for diagnosis of Human SLE^16^. Basophil contribution to such inducible lupus-like model may open new approaches to decipher mechanisms by which basophils interact with the lupus environment to amplify disease. Indeed, the use of mice deficient for some molecular effectors just in the basophil compartment will allow us to pursue this goal.

Here, we analyzed basophil contribution to the pristane-induced LN-like disease in C57BL/6 female mice and showed their accumulation in SLOs where they could support autoantibody-producing plasma cells. Indeed, their depletion by two different means reduced the proportions of these cells along with autoantibody titers and dampened kidney inflammatory status, identifying pristane-induced lupus-like disease as a promising model to study basophil’s involvement in lupus nephritis pathogenesis.

## Results and Discusssion

### Basophils activation and accumulation in SLOs during pristane-induced lupus-like disease

IgE, as the most studied basophil-bound immunoglobulin isotype, regulates basophil activation and ICs-mediated kidney damage in the absence of the low affinity IgG inhibitory receptor (FcγRIIB)^11^; a receptor described to be important in controlling tolerance and autoimmune response^22^. In constitutive Lyn deficiency context, basophils are hypersensitive to FcεRI-mediated stimulation and are hyper-proliferative leading mice to develop a peripheral basophilia. Genetic depletion of this negative regulator mediates also basophil intrinsic overproduction of IL-4, skewing any immunization challenge towards a T_H_2 response and reducing the efficiency of the response to T_H_1-specific disease models^23^. In *Lyn* ^−/−^ mice, an uncontrolled humoral self-response is induced concluding into a lupus-like nephritis phenotype, in which basophils are found to be important contributors in an IgE and IL-4 dependent manner^6^. Importantly, in these spontaneous lupus-like disease, basophils have an activated phenotype and accumulate in SLOs, like what we observed in Human SLE subjects^6^.

**Figure 1:**
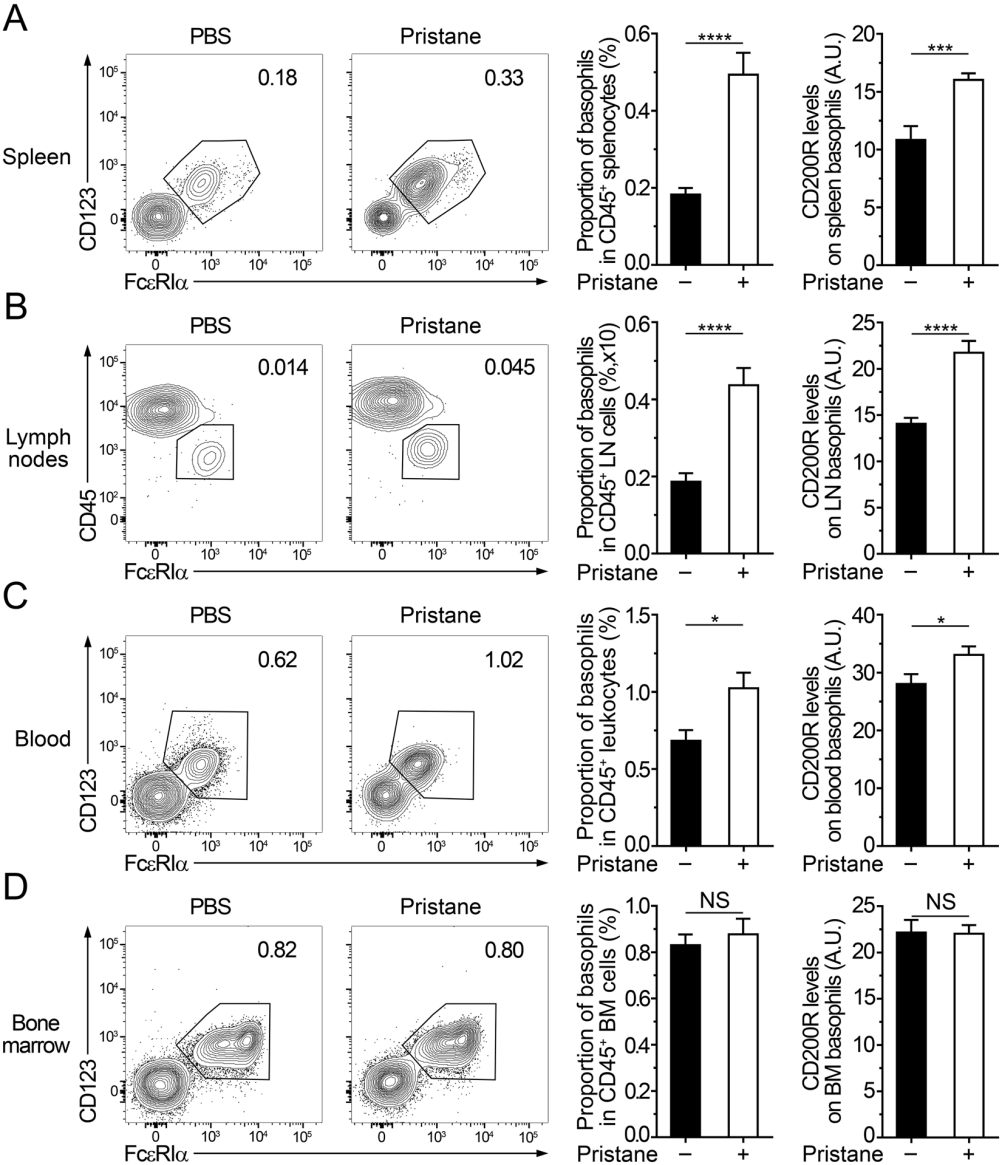
Basophil activation and accumulation in secondary lymphoid organs during pristane-induced lupus-like disease. (**A**–**D**) Contour plots, proportion of basophils among CD45^+^ living cells (defined as CD19^−^ TCRβ^−^ CD117^−^ CD49b^+^ FcεRIα^+^ CD123^+^ CD45^lo^) and CD200R expression levels on basophils (ratio geometric mean of CD200R on basophils on geometric mean of the corresponding isotype control) in spleen (**A**), lymph nodes (cervical, brachial and inguinal) (**B**), blood (**C**) and bone marrow (**D**) from C57BL/6 mice injected intraperitoneally (ip) 24 weeks before the day of experiment with PBS (*n* = 11) or pristane (*n* = 12). Data acquisition was realized by flow cytometry. Data correspond to the pooled results of three independent experiments of at least 3 mice per group. Data are presented as mean + s.e.m. Statistical analyses were by unpaired Student t tests. NS, not significant; *p < 0.05, ***p < 0.001, ****p < 0.0001. A.U.: arbitrary units.

We first analyzed whether such activation and migration to SLOs was observed in the pristane-induced lupus-like model. As hypothesized, an important accumulation of basophils (defined as CD19^−^ TCRβ^−^ CD117^−^ CD49b^+^ FcεRIα^+^ CD123^+^ CD45^lo^ cells) in SLOs of C57BL/6 mice 24 weeks after pristane injection was found (Fig. 1A,B). Furthermore, a peripheral blood basophilia was noticed without any change in bone marrow proportion of basophils as in *Lyn* ^−/−^ mice^23^ (Fig. 1C,D). This latter information suggests that the number of peripheral basophils in the lupus context may be finely regulated in the bone marrow either by an over proliferation of medullar basophil precursors compensated by a faster release in the blood flow or by an extra-medullary differentiation and proliferation of basophils and their precursors^5^. Apart from bone marrow, when compared to PBS injected mice, peripheral basophils from pristane-injected animals had an activated phenotype as measured by the CD200R expression levels on these cells^24^ (Fig. 1A–D).

Altogether, these results showed that during pristane-induced lupus-like disease development, peripheral basophils were activated and accumulated in SLOs, strongly suggesting their contribution to the development of the disease.

### Antibody-mediated versus diphtheria toxin receptor-mediated basophil depletion

MAR-1 is an Armenian hamster monoclonal IgG recognizing the α chain of the FcεRI which has been identified as a potent tool to deplete mouse basophils *in vivo* ^7^. In the *Lyn* ^−/−^ lupus-like mouse model, we previously showed the effects of MAR-1-induced basophil-depletion on disease parameters, demonstrating the contribution of basophils to the support of autoantibody production and kidney inflammation in this model^6^. However, MAR-1 mediated basophil depletion was shown to induce bystander effects such as mast cell and neutrophil transient activation or depletion of a FcεRIα bearing DC subset in the context of house dust mite (HDM) immunization^7, 25, 26^. In recent years, new genetic models of basophil depletion were published and became available to the community^27, 28^. The *Mcpt8* ^*DTR*^ mice allow diphtheria toxin (DT) mediated depletion through the expression of the human DT receptor under the control of the *Mcpt8* gene which expression is basophil-specific and preserved in these mice unlike other basophil-specific mouse model^27–29^. Of note, the latter point has been recently described as central in basophil-regulated immune responses^30^.

**Figure 2:**
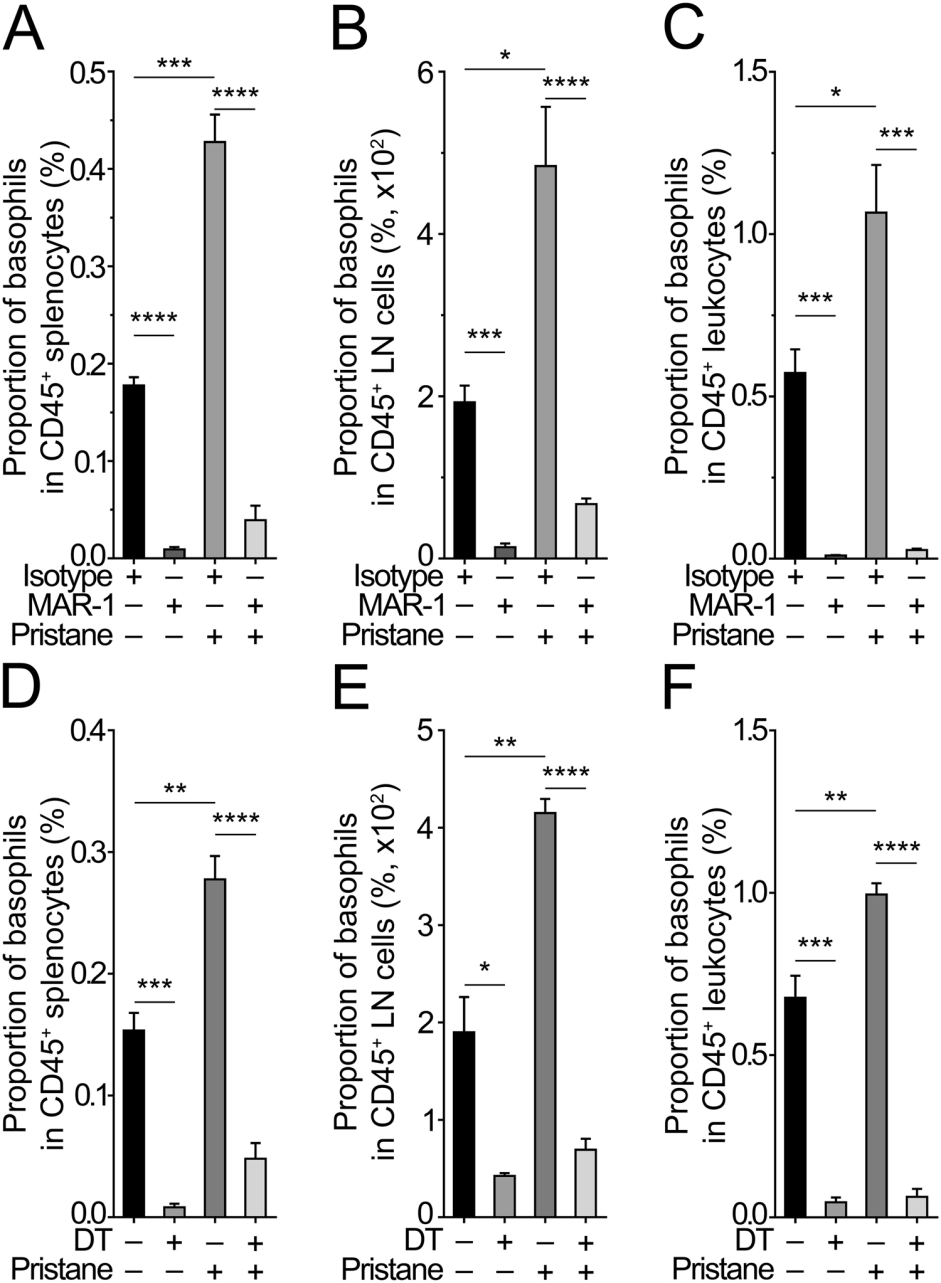
Efficient basophil depletion induced by Mar-1 antibody or diphtheria toxin. (**A**–**F**) Proportion of basophils among CD45^+^ cells (as described in Fig. 1) in spleen (**A**,**D**), lymph nodes (**B**,**E**) and blood (**C**,**F**) as determined by flow cytometry, in mice described thereafter. (**A**–**C**) PBS- or pristane-injected C57BL/6 mice basophil-depleted or not through treatment with MAR-1 antibody or isotype control, respectively, as described in the ***Methods***. (**D**–**F**) PBS- or pristane-injected C57BL/6 *Mcpt8* ^*DTR*^ mice basophil-depleted or not through treatment with diphtheria toxin (DT) or PBS, respectively, as described in the ***Methods***. Data acquisition was realized by flow cytometry. Data correspond to the pooled results of at least three independent experiments. Per group, *n* = *3–8* mice. Data are presented as mean + s.e.m. Statistical analyses were by unpaired Student t tests. NS, not significant; *p < 0.05, **p < 0.01, ***p < 0.001, ****p < 0.0001.

Since pristane treatment induced a peripheral basophilia (Fig. 1), we next tested in these mice whether basophil depletion was still reachable with both MAR-1 and DT approaches. In both case, mice were depleted between the 22^nd^ and the 24^th^ weeks after pristane injection, as described in the ***Methods***, allowing us to analyze the effects of basophil depletion on disease parameters at this time point. Regular C57BL/6 female mice were indeed basophil-depleted in secondary lymphoid organs (spleen and lymph nodes) and blood independently of their pristane or PBS treatments after two consecutive weeks of MAR-1 injections (Figs 2A–C and S1). Similarly, *Mcpt8* ^*DTR*^ female mice (on the same C57BL/6 genetic background) were as well basophil-depleted in secondary lymphoid organs and blood independently of the pristane injection after two consecutive weeks of DT treatment (Figs 2D–F and S1).

These results confirmed that basophil depletion could be reached over a two weeks period of time even in pristane-injected mice despite the peripheral basophilia developed, and that basophil contribution to pristane-induced lupus-like disease may be evaluated with these two distinct basophil-depletion approaches.

### Basophils amplify autoantibody production in pristane-induced lupus-like disease

**Figure 3:**
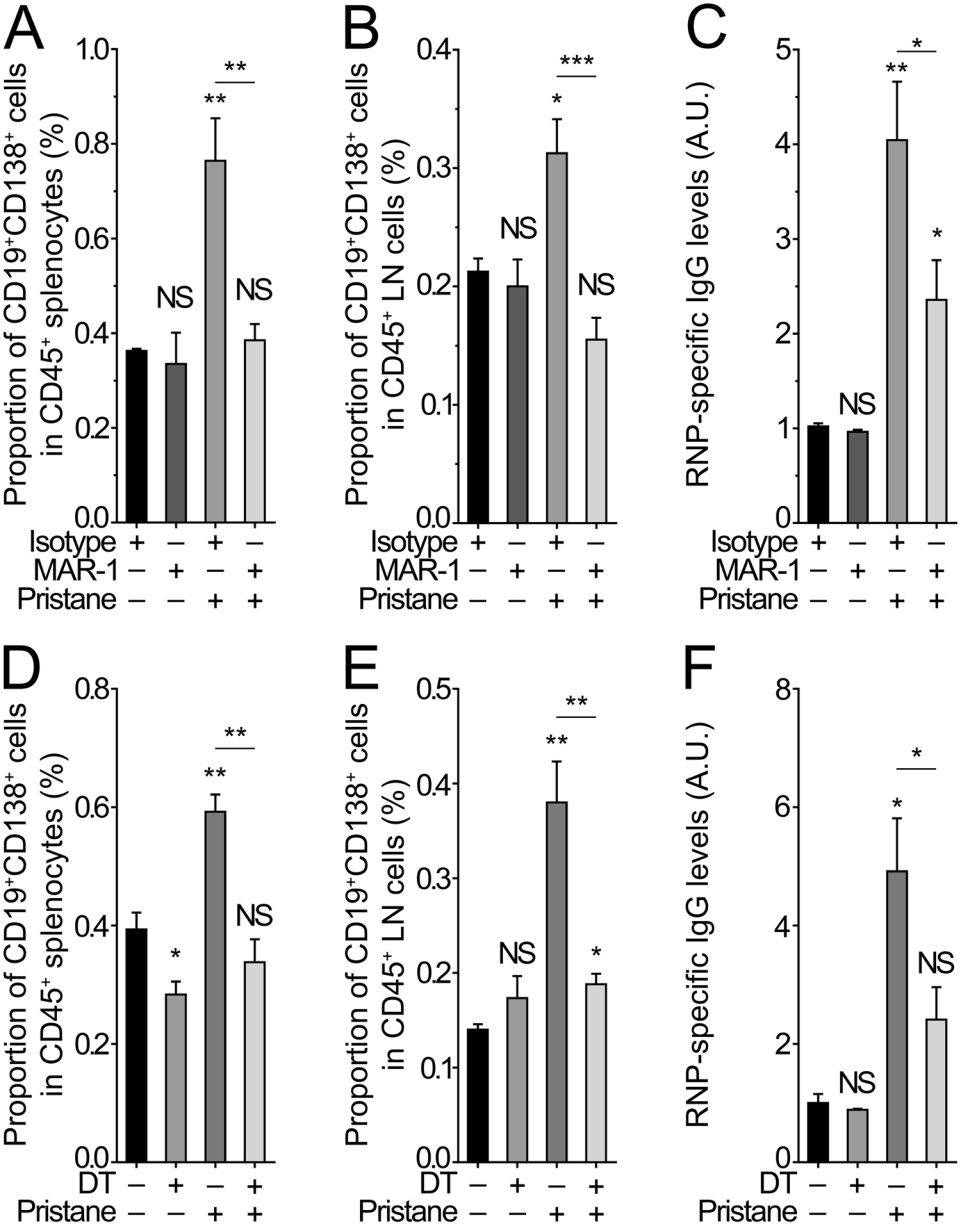
Basophil depletion dampens proportions of short-lived CD19^+^CD138^+^ plasma cells in SLOs and serum autoantibody titers during pristane-induced lupus-like disease. (**A**,**B**,**D**,**E**) Proportion of CD19^+^CD138^+^ cells among living CD45^+^ cells (defined as CD45^+^ TCRβ^-^ CD19^+^ CD138^+^) in SLOs (spleen (**A**,**D**) and lymph nodes (**B**,**E**)) from the same mice as in Fig. 2 and as determined by flow cytometry. (**C**,**F**) Levels of anti-RNP specific IgG in serum from the same mice as in Fig. 2. Optical density values at 450 nm were normalized to mean value of the control group for each set of experiments (PBS-injected basophil-sufficient mice). Data are presented as mean + s.e.m. Statistical analyses were by unpaired Student t tests. NS, not significant; *p < 0.05, **p < 0.01, ***p < 0.001. A.U.: arbitrary units.

Pristane injection induces a polyclonal hypergammaglobulinemia associated with an enrichment of antibody producing cells and high autoantibody titers raised against nuclear antigens (mainly ribonucleoproteins in the C57BL/6 genetic background) leading to the chronic autoimmune phenotype^16, 31^. The autoantibody titers are maintained by the continuous source of necrotic/apoptotic ligands driving cytokine production and B cell differentiation^18, 32^. We confirmed these data by detecting a significant hypergammaglobulinemia and a significant increase in proportion of antibody producing cells (CD19^+^CD138^+^) and in anti-RNP specific IgG autoantibody titers in pristane-injected C57BL/6 mice (Figs S2 and 3). Our group and others have previously shown that basophils support B cell proliferation and directly promote plasma cell survival and Ig production in lupus and normal environments^6–9, 33^. Following both strategies of basophil-depletion, short lived CD19^+^CD138^+^ plasma cell proportions in SLOs were reduced in pristane-injected and basophil-depleted mice (Fig. 3A,B,D,E). As expected, this phenomenon was accompanied by reduced RNP-specific autoantibody serum titers when basophils were absent, independently of the method used to deplete basophils (Fig. 3C,F). Basophil depletion showed as well a clear trend to decrease the pristane-induced hypergammaglobulinemia (Fig. S2).

These results evidenced that basophils contribute directly to the B cell humoral response associated to pristane treatment, that their depletion dramatically reduces the peripheral amounts of pathogenic autoantibodies and strongly suggested that their depletion in the pristane-induced lupus nephritis-like disease may lead to alleviate ICs-mediated kidney inflammation.

### Basophil depletion dampens pristane-induced lupus-like nephritis

**Figure 4:**
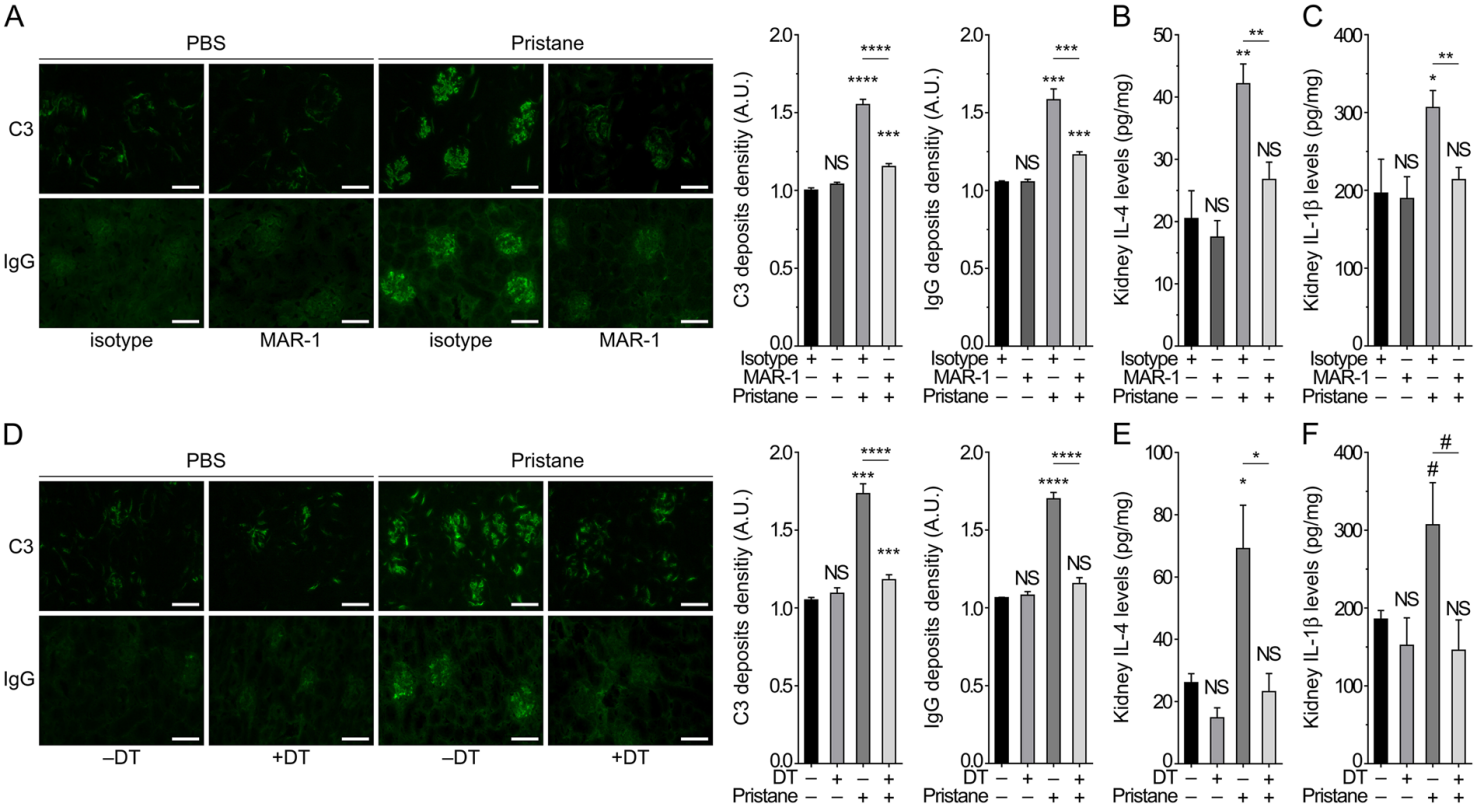
Basophil depletion dampens pristane-induced kidney disease. (**A**,**D**) Representative immunofluorescence staining for C3 and IgG deposits in kidneys from mice treated as indicated and the corresponding quantifications in mice as described in Fig. 2. Scale bar = 500 µm. (**B**,**C**,**E**,**F**) Levels of IL-4 (**B**,**E**) and IL-1β (**C**,**F**) in total kidney protein extracts from mice as described in Fig. 2 and as assessed by ELISA. Data are presented as mean + s.e.m. Statistical analyses were by unpaired Student t tests. NS, not significant; *p < 0.05, **p < 0.01, ***p < 0.001, ****p < 0.0001. ^#^p < 0.1. A.U.: arbitrary units.

We previously demonstrated with the *Lyn* ^−/−^ lupus-like model that MAR-1-mediated basophil depletion could decrease the pro-inflammatory milieu in kidneys from aged and sick *Lyn* ^−/−^ mice^6^. The lupus-like glomerulonephritis induced by pristane in C57BL/6 female mice is described to be mild (mesangial, Class II)^16^. Accordingly, 24 weeks after pristane injection, we observed histologically some kidney lesions (at the glomerular level) associated with an increased albuminuria (Fig. S3). The induced lupus-like nephritis was further evidenced by an accumulation of IgG and complement factor C3 deposits in the glomeruli from pristane-treated mice and by the associated renal inflammation as measured by the increased amounts of the pro-inflammatory cytokines IL-4 and IL-1β in kidney extracts (Fig. 4).

In this pristane-induced lupus-like nephritis, MAR-1-mediated depletion of basophils over a two weeks period of time led to a clearance of C3- and IgG-containing ICs glomerular deposits in the kidney of pristane-injected mice (Fig. 4A). This was associated with a dramatic decrease in kidney IL-4 and IL-1β pro-inflammatory cytokines concentrations (Fig. 4B,C) as we previously demonstrated in the *Lyn* ^−/−^ lupus-like model^6^. MAR-1 injection is described to transiently and moderately activate mast cells, which may lead to a transient and moderate increase in systemic levels of vasoactive compounds such as histamine^7, 28^. The latter point explained the tendency of MAR-1 treated animals, independently of the pristane treatment, to increase their albuminuria (Fig. S3). Importantly, DT-mediated basophil depletion in pristane-injected *Mcpt8* ^*DTR*^ mice over the same period of time led as well to a dampening of ICs glomerular deposits and pro-inflammatory cytokine levels in their kidneys (Fig. 4D–F). This pristane-treated *Mcpt8* ^*DTR*^ mice showed a tendency to normalize their urinary ACR after two weeks of DT-mediated basophil depletion (Fig. S3). Identifying a method to deplete basophils for a longer period of time may be needed to see an improvement at the kidney histological level, along with a significant improvement in kidney function.

Altogether these results demonstrate that basophils contribute to the development of pristane-induced lupus-like nephritis by accumulating in SLOs of pristane-treated animals where they amplify the autoantibody production through the support of CD19^+^CD138^+^ autoantibody producing cells.

Pristane injection is an inducible mouse model of lupus nephritis, due to a chronic accumulation of autoantibodies promoted by the expansion of self-nuclear antigens specific plasma cells^16^. Here, we show that basophils play an important role in the pristane-induced kidney pathology by amplifying the autoantibody production. Both basophil depletion approaches used in our present report allowed to demonstrate the same conclusions about the involvement of basophils in the pristane-induced lupus-like nephritis model. Our study validate basophil targeting as a putative therapeutic approach to improve or prevent lupus nephritis symptoms. Transient basophil depletion in SLE patients may lead to dampen the severity of a flare or prevent it to happen. However, since basophils are important players in immunity against infections^34^, identifying basophil-specific targets allowing to modulate their activity and/or their migration to SLOs without impacting their anti-infectious properties is required. Our results open new ways to study mechanisms by which basophils are accumulating in SLOs and contribute to disease development during lupus pathogenesis. Indeed, the inducible pristane model may now be used in basophil-specific genetically modified mice to identify molecular effectors involved in their contribution, and avoid the need of backcrossing such mice to a genetically-mediated spontaneous lupus-like mouse model background. Hopefully, this more basophil-specific approach in the lupus environment will allow to identify novel therapeutic targets for LN patients.

## Methods

### Mice

8 weeks old female C57BL/6 mice were purchased from Charles River laboratories. *Mcpt8* ^*DTR*^ mice^28^ were on a C57BL/6 genetic background and bred in our pathogen-free animal facility. Mice were maintained following the French and European guidelines and the study was approved by the local ethical committee (comité d’éthique en expérimentation animale, Faculté de Médecine Site Bichat Université Paris Diderot) and by the Department of Research of the French government under the animal study proposal number 02484.01. Mice received a single intraperitoneal injection of 0.5 ml of pristane (Sigma-Aldrich) or phosphate-buffered saline (PBS, Gibco) as a control.

### *In-vivo* basophil depletion

DT-mediated basophil depletion: Female *Mcpt8* ^DTR^ mice (with the C57BL/6 genetic background) received four intraperitoneal injections of 1 µg of diphteria toxin (D05664; Sigma-Aldrich) (or the corresponding vehicle, PBS) at day (D)-13, D-12, D-8 and D-4 before the 24^th^ week after pristane injection was reached (D0).

MAR-1-mediated basophil depletion: Female C57BL6 WT mice were injected retro-orbitally with 20 µg of the anti-FcεRIα antibody (clone Mar-1, BioLegend) or isotype control (Armenian hamster IgG, Innovative Research, Inc) at day (D)-13, D-12, D-10 and D-6, D-5 and D-4 before the 24^th^ week after pristane injection was reached (D0).

### Autoantibody detection

Levels of IgGs anti-RNPs were measured by ELISA with plates coated with purified RNP complex from calf thymus (ImmunoVision) following the previously reported protocol^11^. The detection antibody used was horseradish peroxidase (HRP)-goat anti-mouse IgG (Jackson Immuno Research laboratories) and the colorimetric reaction was visualized by tetramethylbenzidine substrate (ThermoFisher scientific). Optical density at 450 nm was measured by spectrophotometry (Infinite 200 Pro plate reader, TECAN, Männedorf, Switzerland).

### Organ collection and cell isolation

Heparinized whole blood samples were collected by cardiac puncture from CO_2_ euthanized mice. Single cell suspensions from spleen and lymph nodes (cervical, inguinal and brachial) were prepared by mechanical disruption over a 40 µm cell strainer (Falcon). Red blood cells (RBC) from spleen, blood and bone marrow (BM) were lysed with RBC lysing buffer (150 mM NH_4_Cl, 10 mM NaHCO_3_, 2.5 mM EDTA). All cells were re-suspended in FACS buffer (PBS containing 1% Bovine Serum Albumin (BSA) and 0.1%NaN_3_) for FACS analysis. Both kidneys were collected. Left kidney was embedded in O.C.T. freezing medium (CellPath, Ltd), snap frozen in liquid nitrogen and kept at −80 °C for later use. Right kidney was cut into two halves. The first half was homogenized with a homogeniser (Fisher Scientific) in ice-cold PBS containing protease inhibitors (ThermoFisher Scientific), centrifuged 10 minutes at 10,000 g and the supernatant was kept at −80 °C for later detection of cytokine levels. The second half was fixed in 10% formalin (Sigma-Aldrich), paraffin-embedded, cut into 4 µm sections and Masson’s trichrome staining was performed to analyze kidney histology.

### Flow cytometry

Cell were counted with a hemocytometer in Trypan blue and 2 million cells per point were used. Cell suspensions were washed in PBS and stained in 100 µL of a 1:100 dilution of Ghost Violet 510 Viability dye (TONBO bioscience) in PBS in the dark for 25 min at 4 °C. After washing with FACS buffer, cells were incubated in 20 µL of home-made blocking solution containing 100 µg/mL of polyclonal mouse IgGs, polyclonal rat IgGs (Jackson Immunoresearch), 10 µg/mL of Hamster Armenian IgGs (Innovative Research, Inc) and 10 µg/mL of rat anti-mouse CD16/32 (clone 2.4G2, BioXcell). Cells were stained for 30 min at 4 °C in the dark in 200 µL of FACS buffer containing fluorophore-conjugated antibodies, then washed in FACS buffer before data collection.

### Antibodies

Basophil staining panel: FITC anti-mouse CD49b (clone HMα2, BioLegend), PerCP-eFluor® 710 anti-mouse CD200R (clone OX110, eBioscience), Alexa Fluor® (AF) 647 anti-mouse FcεRIα (clone Mar1, BioLegend), AF700 anti-mouse Gr1 (clone RB6–8C5, BioLegend), APC-Cy7 anti-mouse CD19 (clone 6D5, BioLegend), APC-Cy7 anti mouse TCRβ (clone H57–597, BioLegend), APC-Cy7 anti-mouse CD117 (clone 2B8, BioLegend), Pacific Blue® (PB) anti-mouse IA-IE (Clone M5/114.15.2, BioLegend), Brilliant Violet® (BV) BV605 anti-mouse CD11b (clone M1/70, BioLegend), PE anti-mouse CD123 (clone 5B11, BioLegend), PE-Cy7 anti-mouse CD45 (clone 30-F11, BioLegend). B cell staining panel: FITC anti-mouse IgM (clone RMM-1, BioLegend), PerCP-Cy5.5 anti-mouse CD11b (clone M1/70, BioLegend), APC anti-mouse CD138 (clone 281–2, BioLegend), AF700 anti mouse TCRβ (clone H57–597, BioLegend), BV421 anti-mouse CD3ε (clone 145–2C11, BioLegend), BV605 anti-mouse F4/80 (clone BM8, BioLegend), PE anti-mouse CD19 (clone 6D5, BioLegend), PE-Cy7 anti-mouse CD45 (clone 30-F11, BioLegend). For both panels, the corresponding isotype controls were used: FITC Armenian Hamster IgG (clone HTK888, BioLegend), PerCP-eFluor 710 Rat IgG2a (clone eBR2a, eBioscience), APC-Rat IgG2a (clone RTK2758, BioLegend), PerCP-Cy5.5–Rat IgG2b (clone RTK4530, BioLegend), AF647 Armenian Hamster IgG (clone HTK888, BioLegend), FITC Rat IgG2a (clone RTK2758, BioLegend), AF700 Rat IgG2b (clone RTK4530, BioLegend), PE Rat IgG2a (clone RTK2758, BioLegend), APC/Cy7 Rat IgG2a (clone RTK2758, BioLegend), APC/Cy7 Rat IgG2b (clone RTK4530, BioLegend), APC/Cy7 Armenian Hamster IgG (clone HTK888, BioLegend), BV605 Rat IgG2a (clone RTK2758, BioLegend), BV605 Rat IgG2b (clone RTK4530, BioLegend), PB Rat IgG2b (clone RTK4530, BioLegend) and BV421 Armenian Hamster IgG (clone HTK888, BioLegend). FACS data were collected with a LSRII-Fortessa flow cytometer using DIVA software (BD Biosciences) and analyzed with FlowJo v10.0.7 (Treestar).

### Immunofluorescence staining

OCT embedded kidneys were cut in 4 µm thick sections and fixed in acetone. They were blocked with PBS containing 5% goat serum (Sigma-Aldrich) for 1 hour at room temperature, then washed 3 times with PBS containing 1% BSA and 0.1% Tween 20. Staining was achieved after 2 h of incubation with 5 µg/mL AF488 goat anti-mouse IgG (Jackson Immunoresearch laboratories, Inc) or FITC anti-mouse C3 (CEDARLANE) or the corresponding isotype controls: AF488-goat IgG (Jackson Immunoresearch laboratories, Inc), FITC Rat IgG2a (CEDARLANE), respectively. Slides were then mounted in Immuno-mount (Thermo Scientific) and kept overnight at 4 °C. Pictures of kidney tissues were taken using the fluorescence microscope (LEICA DMR, Leica Mycrosystems). Quantifications of C3 and IgG glomerular deposits were realized with the ImageJ (1.47 v) software by calculating the mean ratio of the fluorescence intensity of at least 20 glomeruli per kidney relative to the fluorescence intensity of the background.

### Enzyme-linked immunosorbent assays (ELISA)

Cytokine (IL-1β and IL-4) levels in kidney extracts were measured by ELISA following manufacturer instructions (BioLegend, San Diego, CA). Total protein levels were determined by BCA Protein Assay following manufacturer’s instructions (ThermoScientific). Levels were represented as amount of cytokine relative to total protein concentration in kidney extracts (pg/mg). Total serum IgG levels were measured by ELISA following manufacturer instructions (Bethyl laboratories, Montgomery, TX).

### Urine Albumin to Creatinine Ratio (ACR) measurements

Urine was collected and the albumin concentration was measured with a mouse albumin ELISA (Bethyl laboratories, Montgomery, TX). A creatinine assay (R&D systems, Minneapolis, MN) was used to determine urine creatinine concentrations. Results are expressed as ACR in μg of albumin per mg of creatinine.

### Statistical analysis

After normal distribution testing (Agostino-Pearson or Kolmogorov-Smirnov tests depending on sample size), differences between groups were analyzed by using unpaired Student t-test, unless otherwise indicated. When more of two groups were compared, one-way analysis of variance (ANOVA) test was used before indicated post-tests if significance (p < 0.05) was reached. Data are presented as means + s.e.m. (standard error of mean). All analysis were performed with GraphPad Prism version 6 (La Jolla, CA, USA). NS, not significant; *p < 0.05, **p < 0.01, ***p < 0.001, ****p < 0.0001.

## Electronic supplementary material

Supplementary Material
