# Supplementary material for: Basophils contribute to pristane-induced Lupus-like nephritis model
Source: Sci Rep. 2017 Aug 11;7:7969. doi: 10.1038/s41598-017-08516-7 (PMC5554199; doi:10.1038/s41598-017-08516-7)
Supplement: Supplementary file 1 — Supplementary Material [file 41598_2017_8516_MOESM1_ESM.pdf]

# SUPPLEMENTARY FIGURES

## Basophils contribute to pristane-induced Lupus-like nephritis model

**Authors**: Barbara Dema[1†], Yasmine Lamri[1†], Christophe Pellefigues[1], Emeline Pacreau[1], Fanny Saidoune[1], Caroline Bidault[1], Hajime Karasuyama[2], Karim Sacré[1,3], Eric Daugas[1,4], and Nicolas Charles[1]*.

**Affiliations**: [1]Centre de Recherche sur l'Inflammation, INSERM UMR1149, CNRS ERL8252, Université Paris Diderot, Sorbonne Paris Cité, Faculté de Médecine site Bichat, Laboratoire d'Excellence Inflamex, DHU FIRE, Paris, France. [2]Department of Immune Regulation, Graduate School of Medical and Dental Sciences, Tokyo Medical and Dental University (TMDU), Tokyo 113-8510, Japan. [3]Department of Internal Medicine, [4]Department of Nephrology, Hôpital Bichat, Assistance Publique-Hôpitaux de Paris, Université Paris Diderot, Faculté de Médecine site Bichat, DHU FIRE, Paris, France. †: BD and YL are co-first authors.

***Corresponding Author**: Nicolas Charles, Centre de Recherche sur l'Inflammation, INSERM UMR1149, CNRS ERL8252, Sorbonne Paris Cité, Université Paris Diderot, Faculté de Médecine site Bichat, 16 rue Henri Huchard, 75018 Paris, France. Phone: +33 157277306 E-mail: nicolas.charles@inserm.fr

**SUPPLEMENTARY FIGURE S1**

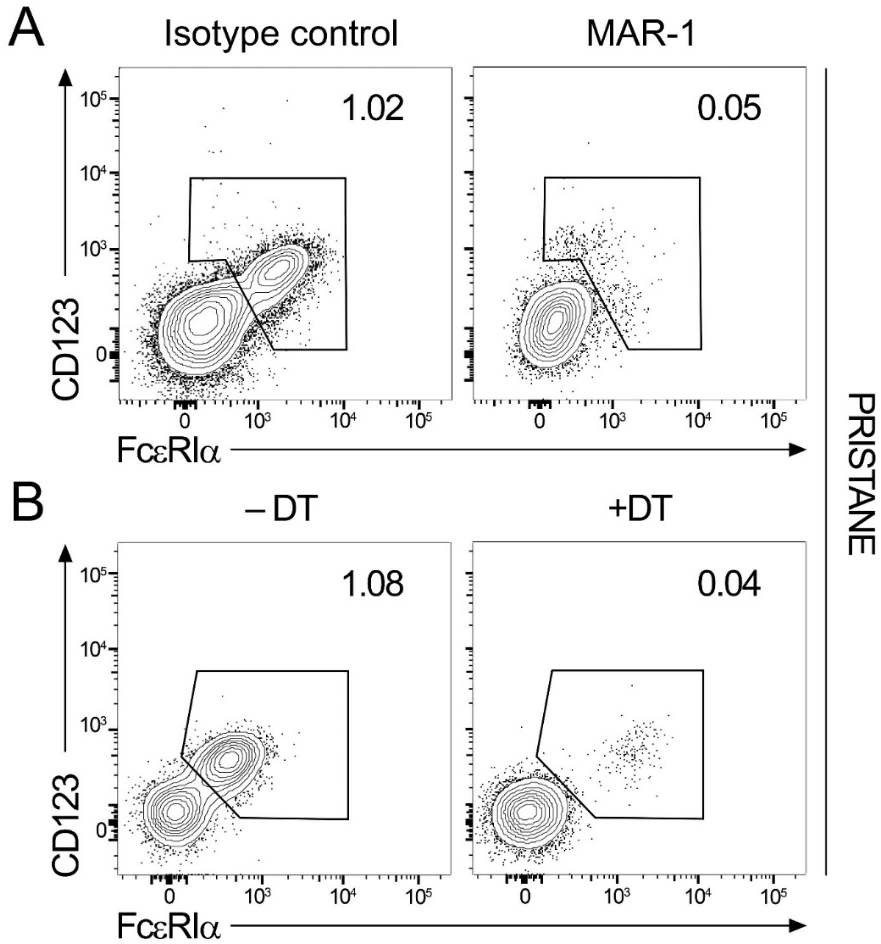


**FIGURE S1. Efficient blood basophil depletion induced by Mar-1 antibody or diphtheria toxin.**

(**A**) Contour plots showing basophils (as defined in **Figure 1**) in the blood of C57BL/6 female mice 24 weeks after pristane injection and treated with the anti-FcεRIα basophil-depleting antibody (MAR-1) or its isotype control, as described in the ***Methods***. (**B**) Contour plots showing basophils (as defined in **Figure 1**) in the blood of *Mcpt8*$^{DTR}$ female mice 24 weeks after pristane injection and treated with diphtheria toxin (+DT) or PBS (–DT), as described in the ***Methods***. (**A**,**B**) Proportion of basophils among singlets living CD45$^{+}$ cells are indicated inside each plot (%).

**SUPPLEMENTARY FIGURE S2**

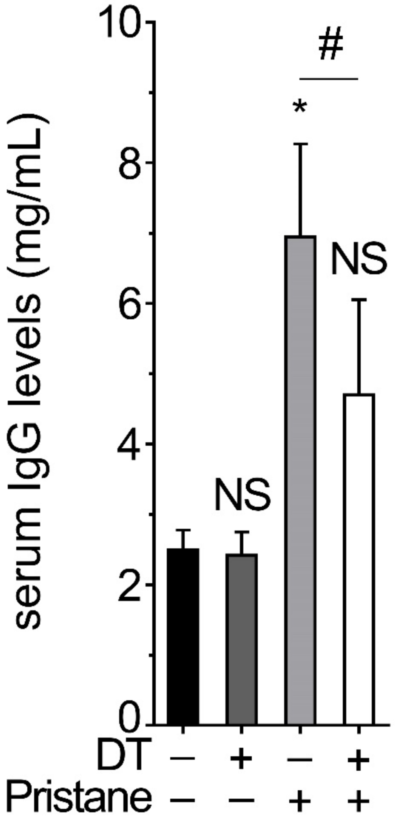


**FIGURE S2. Basophil depletion tends to attenuate pristane-induced hypergammaglobulinemia**.

Levels of IgG in serum from mice as described in **Figure 2D-2F** and as assessed by ELISA. Data are presented as mean + s.e.m.. Statistical analyses were by unpaired Student t tests. NS, not significant; #: $p = 0.1$; *: $p < 0.05$.

**SUPPLEMENTARY FIGURE S3**

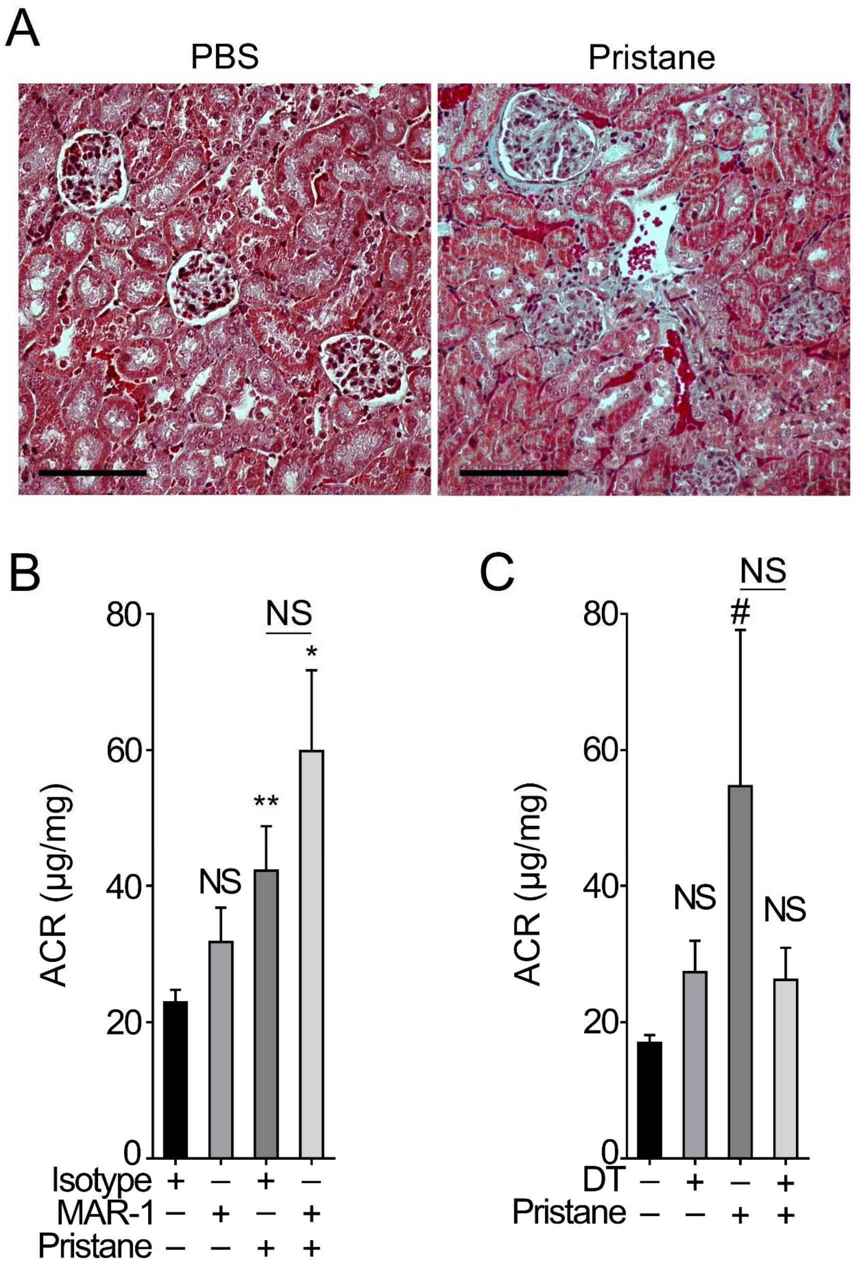


**FIGURE S3. Basophil depletion and pristane-induced kidney lesions**.

(**A**) Masson's trichrome staining of representative kidneys from C57BL/6 mice injected *ip* with PBS (left) or pristane (right) 24 weeks before analysis. Scale bar = 100 µm. (**B**) Albumin to creatinine ratio (ACR) in the urine from mice as described in **Figure 2A-C**, assessed as described in the ***Methods*** section. (**C**) ACR in the urine from mice as described in **Figure 2D-F**, and assessed as described in the ***Methods*** section. (**B**,**C**) Data are presented as mean + s.e.m.. Statistical analyses were by Mann-Whitney tests. NS, not significant; #: $p = 0.1$; *: $p < 0.05$; **: $p < 0.01$.
